# IL-18 and MIG increase one year after transition to dolutegravir-based dual therapy despite sustained viral suppression

**DOI:** 10.3389/fimmu.2026.1784890

**Published:** 2026-05-28

**Authors:** Alexandre Pérez-González, Jacobo Alonso-Domínguez, Inés Martínez-Barros, Aida López-López, Laura Labajo-Leal, Antonio Ocampo, Eva Poveda

**Affiliations:** 1 Infectious Diseases Unit, Department of Internal Medicine, Complexo Hospitalario Universitario de Vigo, Sergas, Vigo, Spain; 2 Virology and Pathogenesis Research Group, Galicia Sur Health Research Institute (IIS Galicia Sur), Sergas-Uvigo, Vigo, Spain; 3 Department of Pharmacy, Complexo Hospitalario Universitario de Vigo, Sergas, Vigo, Spain

**Keywords:** cytokine, HIV, IL-18, inflammation, simplification

## Abstract

**Introduction:**

Modern antiretroviral treatment allowed a significant improvement in the life expectancy of people with HIV (PWH), exhibiting robust and sustained efficacy with scarce toxicities, both in two-drug regimens (2DRs) and three-drug regimens (3DRs). However, a proinflammatory condition is observed even in virologically suppressed PWH. The aim of this study is to assess prospectively the dynamics of various cytokines in PWH who transition from a 3DR to a 2DR.

**Methods:**

A prospective cohort study including 35 virologically suppressed PWH who switched from a 3DR to a dolutegravir-based 2DR was conducted. Plasma concentrations of IL-6, IL-18, IP-10, MIG and IL-18BP were quantified using a multiplex bead-based immunoassay (MILLIPLEX^®^) in samples collected at baseline month 3, month 6 and month 12; an exploratory extension analysis was performed in a subset of 6 participants up to 24 months.

**Results:**

Between June/2021 and December/2022, 35 subjects were recruited, of whom mostly were men (*n* = 25, 71.4%), born in Spain (*n* = 27, 62.8%), with a median time receiving ART of 10.7 years. Throughout the study period no virological failure or transient episodes of low-level viremia were observed. At month 12, no significant changes were observed in total lymphocyte CD4^+^ count (855 c/µL vs 820 c/µL), CD4^+^/CD8^+^ ratio (1.05 vs 1.07), IL-6 (2.24 pg/mL vs 2.24 pg/mL) and IP-10 (188.2 pg/mL vs 245.8 pg/mL). On the contrary, increased plasma concentrations of MIG (2785 pg/mL vs 2966 pg/mL, *p* = 0.017) and IL-18 (63.33 pg/mL vs 75.10 pg/mL, *p* = 0.009) were observed. In this respect, the variations in IL-18 concentrations were not correlated with significant changes concerning IL-18BP (59.26 pg/mL vs 43.34 pg/mL).

**Conclusions:**

At 12 months after switching to a 2DR, plasma IL-18 and MIG concentrations increased, whereas IL-6 and IP-10 remained stable. The rise in IL-18 occurred independently of IL-18BP changes, indicating that reduced IL-18 neutralization is unlikely to explain this finding and supporting the involvement of alternative regulatory pathways.

## Introduction

The introduction of modern antiretroviral therapy (ART) has markedly improved life expectancy among PWH (1). Despite sustained viral suppression, PWH experience a higher burden of non-AIDS comorbidities, such as cardiovascular and metabolic disease, than the general population (2), a phenomenon in which persistent low-grade inflammation and immune activation are thought to play a central role.

Even under suppressive ART, residual immune activation may persist, potentially driven by low-level antigenic stimulation and inflammatory signalling pathways in innate immune cells such as monocytes/macrophages, including inflammasome-related mechanisms (3, 4). In this context, our study group have identified connections between certain proinflammatory cytokines and the immune response to the HIV infection, such as interleukin-18 (IL-18) as a biomarker of durable elite control (5), interferon gamma-induced protein 10 (IP-10; also named C-X-C motif chemokine ligand 10 or CXCL10) and monokine induced by gamma interferon (MIG; also named C-X-C chemokine motif ligand 9 or CXCL9) as sensitive markers of early virological response (6) and also IP-10 and MIG as precocious predictors of loss of control among elite controllers (7).

First-line ART is typically based on an integrase strand transfer inhibitor (INSTI) combined with one or two nucleos(t)ide reverse transcriptase inhibitors (NRTIs), or with a non-nucleoside reverse transcriptase inhibitor (NNRTI). Historically, standard regimens included three active drugs (3DR); however, since the 2010s, two-drug regimens (2DR) have been increasingly adopted, supported by evidence of comparable efficacy and safety to 3DR (8, 9). Whether 3DR provides superior control of subclinical inflammation relative to 2DR remains unresolved, as the available data are heterogeneous and findings to date are conflicting (10, 11).

Randomized switch studies that have compared 2DR with continued 3DR have generally reported no clear, uniform anti-inflammatory advantage of maintaining a third agent, with effects, when present, being small and biomarker-specific. In SWORD-1/2 (switch to dolutegravir/rilpivirine), analyses found no consistent pattern across inflammation or atherogenesis markers overall, although a modest increase in soluble cluster of differentiation 14 (sCD14) at week 48 was observed in at least one analysis (12). In TANGO (switch to dolutegravir/lamivudine) and related pooled analyses, reported changes were similarly subtle (e.g., a modest IL-6 increase with minimal or slightly reduced sCD14), while other markers such as high sensitivity C-reactive protein (hsCRP), soluble cluster of differentiation 163 (sCD163), and D-dimer tended to remain largely stable at week 48 (13).

Accordingly, we conducted a prospective cohort study to characterize the 12-month trajectories of multiple inflammatory biomarkers in individuals who switched from a three-drug regimen (3DR) to a two-drug regimen (2DR). The selection of cytokines was based on our previous studies assessing the roles of these biomarkers in different immunovirological scenarios.

## Material and methods

### Study design

This prospective cohort study assessed the dynamics of several cytokines during a period of 12 months in PLWH who switched from a 3DR to a 2DR, collecting blood samples at baseline, month 3, month 6 and month 12. The study was carried out in the Infectious Diseases Cohort from the IIS Galicia Sur Health Research Institute Biobank (IISGS; B.0000802), comprising PWH receiving care at the Infectious Disease Unit of Álvaro-Cunqueiro Hospital (Vigo, Spain). The recruitment started in June/2021 and ended in December/2022.

### Inclusion and exclusion criteria

Eligible participants were adults (≥18 years) with confirmed HIV-1 infection, virologically suppressed for at least one year, switching from a 3DR to a 2DR during their usual follow-up. The exclusion criteria were designed to reduce possible bias caused by other proinflammatory conditions; therefore, subjects diagnosed of other chronic infections were excluded (i.e., chronic hepatitis B or an active hepatitis C in the last three years). Accordingly, other diseases causing a significant inflammation also constituted an exclusion criteria. This is the case of an active opportunistic infection, active cancer within the 5 years prior to recruitment, autoimmune or autoinflammatory diseases (e.g. systemic lupus erythematosus or inflammatory bowel disease) or an acute infection of any kind at the time of inclusion. In addition, pregnancy and breastfeeding also were considered as exclusion criteria. The recruitment period started in June/2021 and ended in December/2022.

### Definition of variables

Virological suppression was defined as an HIV-RNA ≤ 50 copies/mL, a blip was defined as an HIV-RNA between 50 and 200 copies/mL returning to virological suppression in a second sample. Virological failure was defined as an HIV-RNA above 200 copies/mL. Baseline samples were defined as those obtained prior to the simplification.

### Sample preparation

Peripheral blood samples were obtained by a trained nurse at the Infectious Disease Unit (Hospital Álvaro Cunqueiro, Vigo). For each participant, samples were collected the day before switching from a 3DR to a 2DR (baseline) and subsequently every three months for 12 months. At each visit, two 9-mL EDTA tubes were drawn and transported at room temperature to the IIS Galicia Sur Biobank (IISGS; B.0000802) within 2 hours. Plasma and peripheral blood mononuclear cells (PBMCs) were isolated using Ficoll density-gradient centrifugation according to standardized biobank procedures. Following separation, plasma was aliquoted into pyrogen-free cryovials and stored at −80 °C.

Clinical data (HIV acquisition mode, nadir CD4+ count, clinical history, comorbidities and viral load measurements) were retrieved from electronic medical records.

### Plasma cytokines quantification

Plasma concentrations of IL-6, IL-18, IP-10 (CXCL10) and MIG (CXCL9) were quantified using a multiplex bead-based immunoassay (MILLIPLEX^®^ Human Cytokine/Chemokine Magnetic Bead Panel; Merck KGaA, Darmstadt, Germany). Assays were performed according to the manufacturer’s protocol. The cytokine panel was selected based on prior evidence and preliminary data, prioritizing those markers considered most informative in this context.

Briefly, plasma samples were thawed on ice and vortexing was avoided. Standards, quality controls and plasma samples (run in duplicate) were incubated with magnetic capture beads overnight at 4 °C with gentle agitation. After washing with a magnetic plate washer, detection antibodies and streptavidin–phycoerythrin were added sequentially, and plates were read on the MAGPIX^®^ system (Luminex Corporation, Austin, TX, USA).

Calibration curves were generated using a 5-parameter logistic regression, and analyte concentrations were calculated using xPONENT software. Samples with coefficients of variation (CV) >20% between duplicates were excluded according to predefined quality criteria.

#### IL-18 binding protein quantification by ELISA

IL-18BP plasma concentrations were quantified using a commercially available Human IL-18BP ELISA kit (EHIL18BP; Thermo Fisher Scientific, Waltham, MA, USA), following the manufacturer’s instructions. Plasma samples were diluted 1:3 using Assay Diluent A and analysed in duplicate.

Briefly, diluted plasma samples and standards (0–18, 000 pg/mL) were incubated in 96-well plates pre-coated with anti-IL-18BP monoclonal antibodies. After sequential incubation with a biotin-conjugated detection antibody and streptavidin–HRP, colour development was achieved using TMB substrate. Absorbance was measured at 450 nm using a microplate reader.

IL-18BP concentrations were calculated from a four-parameter logistic standard curve and corrected for sample dilution. The minimum detectable concentration of the assay was 20 pg/mL.

### Data analysis

Categorical variables are presented as frequencies and percentages and quantitative variables as median and interquartile range (IQR). The Shapiro-Wilk test determined non-normal distributions; thus, non-parametric tests were applied. Measurements were performed at baseline, month 3, month 6 and month 12, and longitudinal variations were analysed using the Friedman test for related samples. When significant (p < 0.05), *post-hoc* pairwise comparisons were performed with the Wilcoxon signed-rank test (Bonferroni-corrected). This same procedure was applied to the exploratory analyses of the 18-month and 24-month subsets separately. Baseline comparisons between stage C and non-C groups used the Mann-Whitney U test. Analyses were performed using SPSS Statistics (version 26.0; IBM Corp.), and graphs were created with GraphPad Prism (version 8.0; GraphPad Software).

### Ethical considerations

The study was approved by the Ethics Committee of Galicia (CEIm-G) with code registry 2021/192. All participants provided written informed consent in accordance with the Declaration of Helsinki. Biobank samples and associated data were handled in strict compliance with national regulations on personal data protection and biomedical research. All datasets used for analysis were pseudonymized to ensure participant confidentiality and data security.

## Results

### Clinical and demographic characteristics of the study population

A total of 35 subjects were recruited, of whom most of them were men (*n* = 25, 71.4%), the median of age at the time of recruitment was 48 years and the most frequent ethnicity was Spanish (*n* = 27, 62.8%) followed by Latin-American (*n* = 5, 14.3%). A total of *n* = 12 (34.3%) have suffered an acquired immunodeficiency syndrome (AIDS) defining illness in the past and the median lowest lymphocyte CD4+ count (i.e., nadir) was 180.5 cells/µL. Baseline CD4+ lymphocyte count and HIV-1 viral load at diagnosis were unknown in a half of the study population; among the remaining subjects, baseline CD4+ was 404 c/µL and viral load 89250 copies/mL respectively. The median time living with HIV (i.e., time between HIV diagnosis and recruitment) was 12 years and the median time receiving ART was 10.7 years. The most common acquisition route was men-who-have-sex-with-men (MSM; *n* = 17, 48.6%), followed by heterosexual intercourse (HTX; *n* = 13, 37.1%).

All subjects were virologically suppressed at the time of inclusion, CD4 lymphocyte count was 855 c/µL and CD4/CD8 ratio 1.05. Before the switch all patients received a 3DR; most of them were based on a INSTI plus two NRTIs (*n* = 28; 80%) followed by a NNRTI plus two NRTIs (*n* = 5, 14.3%). Baseline characteristics of the study population are summarized in **Table 1**.

**Table 1:** Baseline characteristics of the study population.

Sex	
Male	25 (71.4%)
Age (years), median (IQR)	48.4 (19.6)
Ethnicity	
Spain, n (%)	27 (77.1%)
Latin-American, n (%)	5 (14.3%)
Other, n (%)	3 (8.5%)
HIV transmission route	
MSM/bisexual, n (%)	17 (48.6%)
Heterosexual, n (%)	13 (37.1%)
IDU, n (%)	5 (14.3)
Prior AIDS-defining illness n (%)	12 (34.3%)
Time living with HIV (years), median (IQR)	12 (8.8)
Time receiving ART (years), median (IQR)	10.7 (9)
T CD4^+^ prior to simplification (cells/µL), median (IQR)	855 (637)
CD4/CD8 ratio prior to simplification, median (IQR)	1.05 (0.73)
Baseline HIV-1 RNA, copies/mL; median	89250
ART prior to simplification	
INSTI plus two NRTIs	28 (80%)
• DTG/3TC/ABC	16
• BIC/FTC/TAF	8
• EVG/c/FTC/TAF	2
• DTG + FTC/TDF	2
NNRTI plus two NRTIs	5 (14.3%)
• EFV/FTC/TDF	2
• NVP + 3TC/ABC	2
• RPV/FTC/TDF	1
PI plus two NRTIs	2 (5.7%)
• ATV + 3TC/ABC	1
• DRV/c/FTC/TAF	1

MSM: men who have sex with men, IDU: injecting drug user, AIDS: acquired immunodeficiency syndrome, ART: antiretroviral treatment, INSTI: integrase strand transfer inhibitor, NRTI: nucleoside reverse transcriptase inhibitors, NNRTI: non- nucleoside reverse transcriptase inhibitor, PI: protease inhibitor, DTG: dolutegravir, 3TC: lamivudine, ABC: abacavir, BIC: bictegravir, FTC: emtricitabine, TAF: tenofovir-alafenamide, EVG: elvitegravir, c: cobicistat, TDF: tenofovir-disoproxil-fumarate, EFV: efavirenz, NVP: nevirapine, RPV: rilpivirine, ATV: atazanavir, DRV: darunavir.

### Clinical and virological outcomes

The most prescribed 2DR was the combination of dolutegravir plus lamivudine in a fixed dose pill (DTG/3TC, Dovato^®^) accounting 29 subjects. In the remaining patients (*n* = 6) the combination of dolutegravir and rilpivirine in a fixed dose pill (DTG/RPV, Juluca^®^) was administered. The prescription of DTG/3TC or DTG/RPV was decided by the HIV physician according to clinical criteria (e.g. DTG/RPV was not prescribed in patients receiving proton bomb inhibitors such as omeprazole owed to drug interactions with rilpivirine).

Throughout the study period two subjects moved to another city thus leaving the study. Both were virologically suppressed after the simplification and before abandoning the study. Among the remaining population, all 33 subjects completed the study period, maintaining viral suppression at month 12. Of note, one subject switch after month 12 from DTG/RPV to intramuscular long acting cabotegravir plus RPV. There were not significant changes in the CD4+ lymphocyte count (855 vs 820, *p* = 0.178) nor CD4/CD8 ratio (1.05 vs 1.07, *p* = 0.805). No side effects attributable to HIV treatment were observed.

#### Dynamics of proinflammatory cytokines and extended follow-up

**Figure 1:**
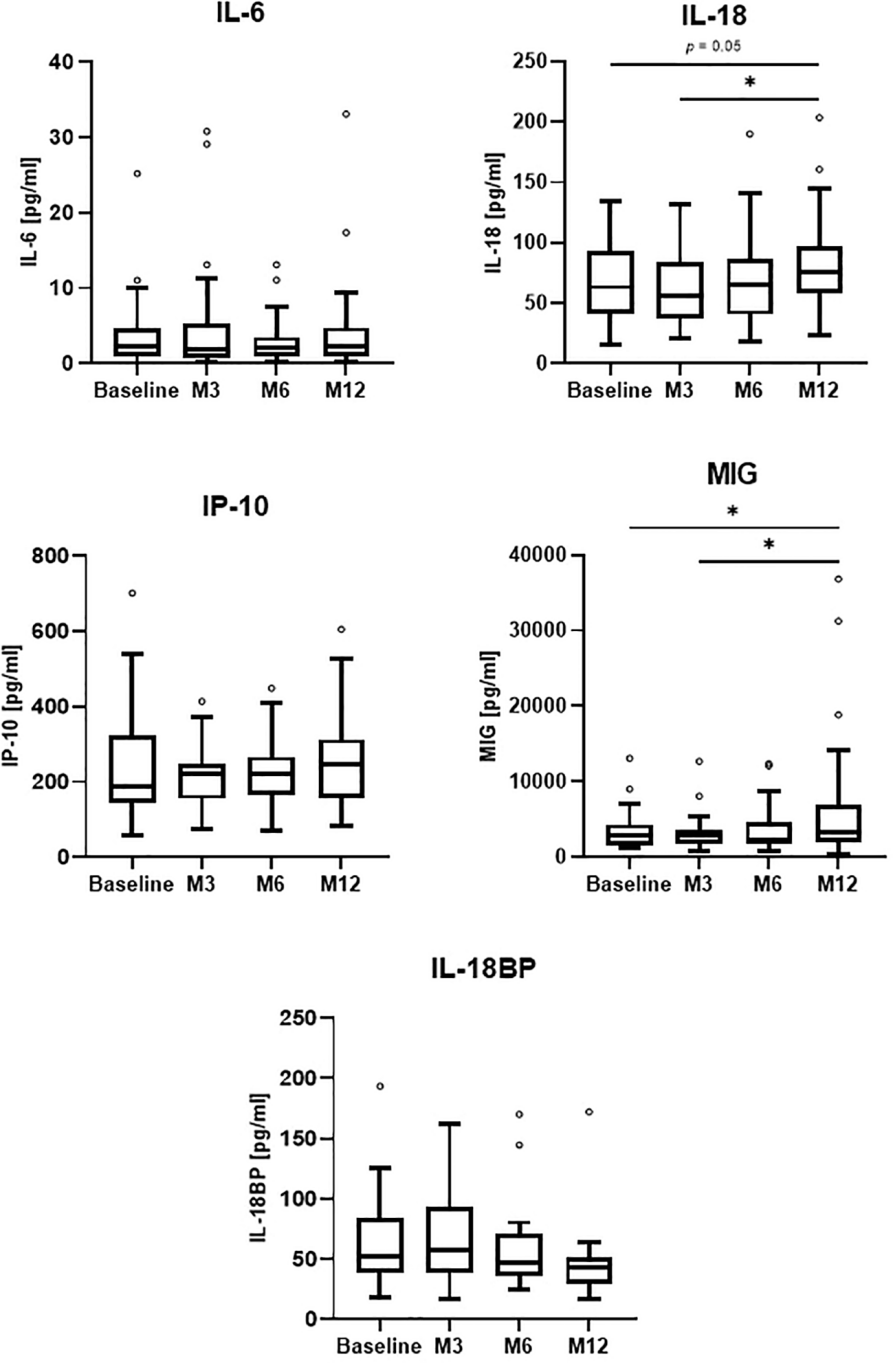
Dynamic changes in plasma IL-6, IL-18, IP-10, MIG, and IL-18BP over 12 months after switching to a two-drug regimen.

During the first 12 months of follow-up, plasma concentrations were assessed at baseline, month 3, month 6 and month 12; the trajectories of these biomarkers are shown in **Figure 1**. The plasma concentrations of IL-6 and IP-10 did not differ between baseline and month 12 (IL-6: 2.24 vs 2.24, *p* = 0.577; IP-10: 188.2 vs 245.8, *p* = 0.427). In contrast, MIG and IL-18 levels increased at month 12 (MIG: 2785 vs 2966, *p* = 0.017; IL-18: 63.33 vs 75.10, *p* = 0.009). Given the observed rise in IL-18, we additionally quantified IL-18 binding protein (IL-18BP), a major endogenous antagonist of IL-18; IL-18BP concentrations showed a non-significant decrease (59.26 vs 43.34, *p* = 0.162).

Because of the changes observed in MIG and IL-18 at month 12, we conducted an exploratory extension of the study using stored samples from the IISGS biobank, assessing the same biomarkers at months 18 and 24. Only 6 participants had samples available for this extended analysis. In this subgroup, IL-18 remained higher at month 24 compared with baseline (83.8 vs 105.8, *p* = 0.003), whereas MIG declined over the same timeframe (3107 vs 1812, *p* = 0.019). IL-6 and IP-10 showed non-significant upward trends (IL-6: 2.15 vs 9.32, *p* = 0.085; IP-10: 212.1 vs 372.8, *p* = 0.051), and IL-18BP did not show significant variation during the extension phase (all comparisons *p*>0.05).

#### Cytokine trajectories among participants with a prior AIDS-defining condition

We compared plasma biomarker concentrations between participants with a history of an AIDS-defining condition (CDC stage C; *n* = 12) and those without (CDC stages A–B; *n* = 23). At baseline, current CD4+ T-cell counts (827 vs 878 cells/µL, *p* = 0.319) and CD4/CD8 ratios (0.89 vs 1.08, *p* = 0.180) were comparable between groups. Baseline plasma concentrations of IL-6 (2.21 vs 3.08, *p* = 1.000), IL-18 (55.66 vs 80.45, *p* = 0.501), IP-10 (183.3 vs 193.1, *p* = 0.601), and IL-18BP (44.26 vs 66.09, *p* = 1.000) were also similar. Likewise, at month 12, these biomarkers remained comparable between subgroups.

In contrast, MIG concentrations were consistently higher in the CDC stage C subgroup both at baseline (3733 vs 196.4, *p* = 0.006) and at month 12 (4597.3 vs 2065.1, *p* = 0.022).

## Discussion

Subclinical inflammation is a major contributor to the development of non-AIDS comorbidities among PWH. Consequently, delineating the mechanisms that sustain immune activation despite virological suppression, and characterizing the longitudinal behavior of key mediators, remains clinically relevant. Multiple drivers of persistent inflammation have been proposed, including altered monocyte metabolism, residual HIV-1 transcription, macrophage activation, and dysregulated intercellular communication mediated by extracellular vesicles (4, 14–18).

Within this framework, whether standard three-drug regimens (3DR) control low-grade inflammation more effectively than two-drug regimens (2DR) remain unsettled, with inconsistent findings across the literature (10, 11). Importantly, available evidence is heterogeneous, encompassing cross-sectional analyses, retrospective and prospective cohorts, and randomized clinical trials, with substantial variability in study populations, ART histories, and biomarker panels. This methodological diversity likely contributes to the discrepant results reported to date.

This study provides valuable information concerning the IL-18 axis, a novel approach to understand the regulation of the immune response to HIV, and the role of the inflammasome in the pathogenesis of several comorbidities. However, analyzing the interactions between HIV and the immune system is challenging owing to the vast number of cell types, soluble mediators and genes involved. In addition, the inflammatory routes exhibit several intersections and different mechanistic effects according to many factors, such as tissue-specific conditions, HIV genetic variability, or the host genome. In this respect we selected a cytokine panel according to the available data and our previous studies focused on IP-10 and MIG. Finally, we decided to explore IL-18 and its main antagonist IL-18BP owing to its role as mediators of interferon gamma (IFNγ) production, a major player in the pathogenesis of non-AIDS comorbidities such as atherogenesis, vascular fibrosis or neuropathic damage (19, 20). Firstly, we assessed IL-6, a pleiotropic cytokine centrally involved in chronic inflammation and immune activation (21). Due to its relevance in the immune response, IL-6 has been extensively examined in PWH, with special interest in its possible relationship with ART modifications. In this respect, whether 2DR causes higher production of IL-6 compared to more intense ART was dismissed in several clinical trials. The SALSA randomized trial, which compared switching to dolutegravir/lamivudine (DTG/3TC) versus continuing a 3- or 4-drug regimen, found no between-group differences in IL-6 at week 48, and similarly no differences in CD4+ T-cell counts or CD4/CD8 ratio (22). Consistent results were reported in the TANGO trial at week 144 among participants switching from a tenofovir alafenamide (TAF)-based regimen to DTG/3TC (13). Likewise, the SWORD-1 and SWORD-2 trials, evaluating a switch to DTG/rilpivirine (DTG/RPV), reported stable IL-6 concentrations through week 148 (12). In line with these studies, among our study population IL-6 remained stable from baseline to month 12 (2.24 vs 2.24, *p* = 0.577). Other observational data, including that from Lombardi et al, also support the absence of meaningful IL-6 changes after switching (23).

Consecutively, we assessed other cytokines also involved in the immune response against HIV. This is the case of IP-10 and MIG, two cytokines closely related to the monocyte macrophage complex and IFNγ production. The disfunction of monocyte metabolism and overproduction of IFNγ have been identified as major causes of cell exhaustion, premature cell death and increased fibroblast activity, ultimately leading to atherosclerosis and premature senescence (24). Previous data shows that IP-10 (CXCL10), an IFN-γ–inducible chemokine produced by multiple cell types, is consistently linked to HIV pathogenesis (25) and often reflects viral replication dynamics: it is elevated in untreated infection, correlates positively with HIV-RNA, and inversely with CD4+ T-cell counts. Following ART initiation, IP-10 typically declines substantially, although levels may remain above those observed in HIV-uninfected individuals (26). In our cohort, IP-10 did not change significantly over 12 months after switching to a 2DR (188.2 vs 245.8, *p* = 0.427), in line with the prospective observational cohort reported by Vassallo et al., where IP-10 remained stable at months 18–24 after simplification (27).

On the other hand, MIG (CXCL9) is a chemokine implicated in immune cell trafficking, differentiation, and activation (28). Persistent monocyte/macrophage dysfunction during HIV infection has been associated with increased MIG production even under virological suppression (17). MIG is characteristically increased in untreated infection, particularly in individuals with high viral load and low CD4+ T-cell counts, and decreases after ART initiation (6, 29, 30).Although SALSA and TANGO did not report IP-10 or MIG outcomes, limiting direct cross-trial comparisons, our findings provide additional insight: MIG rise modestly at month 12 (2785 vs 2966, *p* = 0.017), but decreased at month 24 in the exploratory extension (3107 vs 1812, *p* = 0.019). Given that the extension analysis was restricted to 6 participants with stored samples, these longitudinal observations should be interpreted cautiously. In addition to the immune signalization, MIG induces the expression of nociceptive channels in primary sensory neurons, suggesting a possible correlation between MIG and HIV-associated peripheral neuropathy (19).

Importantly, our group and others have shown that IP-10 and MIG track HIV replication and its suppression: both are high in treatment-naïve infection, decline with ART in parallel with HIV-RNA decay (6), and increase in elite controllers who subsequently lose virological control (7). In this context, the absence of significant changes in IP-10 after switching to a 2DR, together with the lack of a consistent increase in MIG over time in the available longitudinal data, does not support a clear signal of enhanced HIV-driven immune activation following regimen simplification. Nevertheless, because we did not assess residual viremia with ultrasensitive virological methods, the contribution of low-level viral transcription or intermittent residual replication cannot be excluded, and the observed chemokine patterns should be interpreted as indirect evidence only.

Finally, we assessed IL-18 and its counterbalance IL-18BP, an axis involved in the activation of the inflammasome nuclear factor kappa-light-chain-enhancer of activated B cells (NFκβ). The relationship between IL-18 and several immune diseases is well documented (e.g., Still’s disease and systemic juvenile idiopathic arthritis) (31), although its relationship with HIV infection is less studied. IL-18, a member of the IL-1 cytokine superfamily, is primarily produced by macrophages following inflammasome activation (notably NLRP3) (32). In conjunction with IL-12, IL-18 promotes IFN-γ production, amplifying proinflammatory signaling. IL-18BP is the principal endogenous inhibitor of IL-18, binding IL-18 and preventing receptor engagement, thereby limiting downstream inflammation (33). In HIV infection, IL-18 concentrations are generally higher than in uninfected controls and have been linked to higher viral load and disease progression (34). Several HIV components have been proposed to trigger inflammasome activation, contributing to IL-18 release (35). Conversely, IL-18BP has been reported to be reduced in PWH, potentially creating a relative excess of bioactive IL-18 (36). ART initiation is associated with decreases in IL-18, particularly among virologically suppressed individuals (37), even though we previously reported higher plasma levels of extracellular vesicles containing IL-18 in elite controllers who maintain a long-term ART-free virological response (5). Several factors may induce dysregulations in the IL-18 and IL-18BP axis, such as circulating pathogen associated molecular patterns (PAMPs) or Fas ligand. The interaction between the T-cell receptor (TLR) and PAMPs or Fas stimulates the expression of the IL-18 gene, leading to an increase in pro-IL-18, the IL-18 protein precursor (38). In addition, several HIV components such as the viral protein R (Vpr) (39) or the Tat protein (40) cause mitochondrial damage, producing excessive amounts of reactive oxygen species (mROS), further stimulating the IL-18 production through activation of major transcription factors such as NF-κβ (41). In our cohort, IL-18 increased from baseline to month 12 (63.33 vs 75.10, *p* = 0.009), whereas IL-18BP remained unchanged (59.26 vs 43.34, *p* = 0.162). As SALSA and TANGO did not evaluate IL-18 or IL-18BP, these data provide longitudinal insight into this pathway in the context of DTG-based 2DR simplification. Comparison with SALSA and TANGO beyond IL-6 is also limited, as neither study has published data on IL-18 or MIG. Therefore, it remains unclear whether differences across studies may reflect biological variation, differences in sampling time points or assay methods, or simply the absence of evaluation or reporting of these biomarkers. Notably, the IL-18 rise occurred without evidence of reduced IL-18BP availability, suggesting that factors beyond impaired neutralization, such as sustained inflammasome activation, higher concentrations of mROS, PAMPs and/or altered macrophage responsiveness or upregulated inflammasome NF-κβ may underlie increased IL-18 production after simplification. From a biological perspective, this pattern may also be relevant because the IL-18:IL-18BP ratio could better reflect relative IL-18 bioactivity than absolute IL-18 concentration alone. In this context, the absence of a parallel increase in IL-18BP may be consistent with relatively increased IL-18 availability, although the clinical implications of this finding remain to be clarified.

Given IL-18’s role in pyroptosis, which contributes to CD4+ T-cell depletion and chronic inflammation within lymphoid tissues (42) this modest increase highlights the relevance of residual inflammatory activity that may not be captured by markers such as IL-6 or IP-10 and that is linked to accelerated cardiovascular risk in PWH (43). The clinical significance of higher concentrations of IL-18 is not yet fully elucidated, but growing evidence suggests a correlation between IL-18 and insulin resistance, a major cause of type 2 diabetes mellitus (44). Urinary excretion of IL-18 has also been linked to higher glycated hemoglobin and chronic kidney disease (45).

It should be noted that this study included PWH under two distinct companion drugs, 3TC and RPV, although the sample size did not allow to explore differences between these two regimens. At the moment, no head-to-head comparison has assessed whether 3TC or RPV have different influence in subclinical inflammation or clinical outcomes. In this respect, RPV could alleviate liver fibrosis, a major player in the development of cirrhosis, through an increased activation of signal transducer and activator of transcription 1 (STAT-1) (46).

This study has limitations. First, the sample size was limited, likely reflecting stringent inclusion/exclusion criteria and the demands of prospective follow-up. Second, as an observational cohort, the study lacked a contemporaneous randomized 3DR comparator arm Despite all available evidence, whether the subclinical inflammation may differ between PWH receiving 2DR and 3DR remain controversial. The major simplification clinical trials did not explore the IL-18 axis; thus, a large gap exists in this matter. Since this study was an observational cohort without a 3DR control arm, we cannot preclude causal inference. Novel randomized clinical trials including IL-18 axis would provide valuable information.

Indeed, other potentially relevant confounding factors were not explored, including cytomegalovirus (CMV) or Epstein-Barr virus (EBV) reactivation, as well as ART adherence. CMV reactivation during HIV infection has been associated with increased production of proinflammatory cytokines including IL-6 or the tumor necrosis factor (TNF) and with clonal expansion of NK cells, a process linked to immune senescence (47). In addition, suboptimal adherence to ART may contribute to low-level viremia, which is a major driver of persistent inflammation (48).

Conversely, strengths include the prospective design and the selection of a well-characterized cohort with exclusion of major confounders that could independently influence inflammatory biomarkers (e.g., relevant inflammatory conditions, coinfections, or malignancies).

In summary, switching to a dolutegravir-based 2DR did not result in significant 12-month changes in IL-6 or IP-10. By contrast, IL-18 increased modestly without a parallel change in IL-18BP, pointing to mechanisms beyond altered IL-18 neutralization as potential drivers of this pattern. MIG also rise at month 12, although this increase was not sustained in the exploratory extended follow-up. The divergent pattern observed during follow-up, with persistently elevated IL-18 but declining MIG levels, suggests that these biomarkers may reflect partially distinct inflammatory processes and highlights the need for further mechanistic investigation.

## Data Availability

The datasets presented in this article are not readily available because the datasets during and/or analysed during the current study available from the authors on reasonable request. Requests to access the datasets should be directed to eva.poveda.lopez@sergas.es.
